# Supplementary material for: Multi‐dataset identification of innovative feature genes and molecular mechanisms in keratoconus
Source: J Cell Mol Med. 2024 Sep 19;28(18):e70079. doi: 10.1111/jcmm.70079 (PMC11412914; doi:10.1111/jcmm.70079)
Supplement: Supplementary file 2 — Material S2. [file JCMM-28-e70079-s002.zip › 7.GSEA analysis/4.MYRF/KEGG_STEROID_BIOSYNTHESIS.html]

Details for gene set KEGG\_STEROID\_BIOSYNTHESIS[GSEA]

|  || Dataset | MYRF.group.cls#h\_versus\_l |
| Phenotype | group.cls#h\_versus\_l |
| Upregulated in class | h |
| GeneSet | KEGG\_STEROID\_BIOSYNTHESIS |
| Enrichment Score (ES) | 0.28299564 |
| Normalized Enrichment Score (NES) | 0.893761 |
| Nominal p-value | 0.5785124 |
| FDR q-value | 1.0 |
| FWER p-Value | 1.0 |
Table: GSEA Results Summary

  

Fig 1: Enrichment plot: KEGG\_STEROID\_BIOSYNTHESIS      
 Profile of the Running ES Score & Positions of GeneSet Members on the Rank Ordered List

  

| SYMBOL | TITLE | RANK IN GENE LIST | RANK METRIC SCORE | RUNNING ES | CORE ENRICHMENT || 1 | CEL | na | 4729 | 0.230 | -0.0185 | Yes |
| 2 | SC5D | na | 4741 | 0.230 | 0.0988 | Yes |
| 3 | LSS | na | 7679 | 0.147 | 0.0891 | Yes |
| 4 | SOAT2 | na | 8341 | 0.131 | 0.1368 | Yes |
| 5 | TM7SF2 | na | 8994 | 0.116 | 0.1773 | Yes |
| 6 | DHCR7 | na | 9487 | 0.106 | 0.2173 | Yes |
| 7 | DHCR24 | na | 11032 | 0.078 | 0.2125 | Yes |
| 8 | SQLE | na | 11767 | 0.067 | 0.2255 | Yes |
| 9 | EBP | na | 11942 | 0.064 | 0.2533 | Yes |
| 10 | CYP51A1 | na | 12032 | 0.063 | 0.2830 | Yes |
| 11 | NSDHL | na | 15012 | 0.028 | 0.2114 | No |
| 12 | MSMO1 | na | 16866 | 0.010 | 0.1630 | No |
| 13 | FDFT1 | na | 18849 | -0.009 | 0.1103 | No |
| 14 | HSD17B7 | na | 20444 | -0.027 | 0.0782 | No |
| 15 | LIPA | na | 20713 | -0.030 | 0.0858 | No |
| 16 | SOAT1 | na | 31531 | -0.292 | -0.0765 | No |
| 17 | CYP27B1 | na | 32207 | -0.330 | 0.0726 | No |
Table: GSEA details [plain text format]

  

Fig 2: KEGG\_STEROID\_BIOSYNTHESIS      
 Blue-Pink O' Gram in the Space of the Analyzed GeneSet

  

Fig 3: KEGG\_STEROID\_BIOSYNTHESIS: Random ES distribution      
 Gene set null distribution of ES for **KEGG\_STEROID\_BIOSYNTHESIS**
